# Mast Fruiting Is a Frequent Strategy in Woody Species of Eastern South America

**DOI:** 10.1371/journal.pone.0001079

**Published:** 2007-10-24

**Authors:** Natalia Norden, Jérôme Chave, Pierre Belbenoit, Adeline Caubère, Patrick Châtelet, Pierre-Michel Forget, Christophe Thébaud

**Affiliations:** 1 Laboratoire Evolution et Diversité Biologique, UMR 5174 Université Paul Sabatier/CNRS, Toulouse, France; 2 Département Ecologie et Gestion de la Biodiversité, UMR 5176 CNRS-MNHN, Brunoy, France; 3 Station d'Etudes des Nouragues, CNRS Guyane UPS 2561, French Guiana, France; University of Sheffield, United Kingdom

## Abstract

**Background:**

It is thought that mast seeding is a rare reproductive strategy in the tropics, since tropical climates are less variable, and fruit consumers tend to be more generalist in these regions. However, previous tests of this hypothesis were based on only few tropical datasets, and none from tropical South America. Moreover, reproductive strategies have been quantified based on the coefficient of variation of interannual seed production, an index that potentially confounds masting and high interannual variability in seed production.

**Methodology/Principal Findings:**

We developed a new approach to model the monthly variability in seed production for 28 tree species, and 20 liana species monitored during 5 years in a tropical forest of Central French Guiana. We found that 23% of the species showed a masting pattern, 54% an annual fruiting pattern, and 23% an irregular fruiting pattern. The majority of masting species were trees (8 out of 11), most of them animal-dispersed. The classification into reproductive strategies based on the coefficient of variation was inconsistent with our results in nearly half of the cases.

**Conclusions/Significance:**

Our study is the first to clearly evidence the frequency of the masting strategy in a tropical forest community of Eastern South America. The commonness of the masting strategy in tropical plants may promote species coexistence through storage dynamics.

## Introduction

Among the wide array of reproductive strategies displayed by plants, mast seeding, the supra-annual and synchronized production of large seed crops, is the most remarkable [1]. Mast seeding has a strong influence on temporal variation in the recruitment of plant populations [2]–[5]. Its occurrence in plant communities is thus expected to have critical implications for population dynamics, and to play a role in the maintenance of species diversity. If seed production is synchronized within species but independent among species, temporal fluctuation in reproduction should allow temporal niche partitioning and promote species coexistence [6]–[7]. In addition, the co-occurrence of mast seeding with other reproductive strategies may increase temporal variation in seed production and thus enhance species coexistence through a storage dynamics effect. In tropical forests, where phenological patterns are diverse [8], the co-occurrence of different reproductive strategies may thus contribute to diversity maintenance.

While tropical forests have provided the most striking examples of mast seeding worldwide [9]–[12], this reproductive strategy is thought to be rare among tropical plant species. Kelly and Sork [1] hypothesized that tropical environments are less likely to select for supra-annual fruiting since these environments are characterized by high plant productivity, low year-to-year climatic variability, and biotic pollination and dispersal–both selecting against masting. Literature compilations on worldwide plant reproduction patterns have shown that variability in annual seed production is inversely correlated with latitude, therefore providing support to Kelly and Sork's hypothesis [1], [13]. However, these studies comprise a disproportionate number of temperate species in the Fagaceae (*Fagus*, *Quercus*) and Pinaceae (*Pinus*), and they include very few tropical datasets. In a recent reappraisal of this question in a tropical forest of central Panama, Wright *et al*. [5] found support for the hypothesis that tropical forests have lower interannual variation in seed production than temperate forests. However, their results also suggest that as much as 50% of the studied species could be classified as masting. Thus, it remains unclear whether mast seeding is a frequent strategy in tropical forests. Additional seedfall studies in other forests of tropical South America and Africa are much in need to address this issue.

Current approaches for assessing reproductive strategies have long been a subject of debate [14], [15]. Most studies have used the coefficient of variation (CV) of interannual seed production to quantify the degree of masting–the standard deviation of annual seed crop divided by the mean of the annual seed crop [1], [5], [13]–[18]. The CV has the advantage of being a dimensionless number, which allows for a comparison among species with significantly different mean and standard deviation values. Annually fruiting species have a low CV, whereas species with a CV typically greater than one are thought of as being mast seeders [14]. However, Herrera *et al*. [15] argued that this index is unable to discriminate between high interannual variability in seed production and masting, since most species show natural year-to-year variation in seed output, which may result in high CV values. If masting is an extreme form of high year-to-year variation in seed output, erratic phenological patterns do not necessarily imply the notion of masting. In addition, the CV ignores the intra-annual variability in seed production: two species with the same number of fruiting events and the same amount of seed output per event will have the same CV irrespective of the temporal sequence of these fruiting events. If we are to estimate with greater accuracy the frequency of species displaying a mast seeding behavior, we need a quantitative framework in which all components of temporal variability in seed production are taken into account.

In this paper, we present the first long-term (5-year) community-wide seedfall dataset in a South American old-growth tropical forest. To assess the interannual variation in seed production across 48 woody species, we develop a novel approach to quantify temporal variation in seed production patterns both within and across years. This novel method provides quantitative parameters that help define masting using a set of objective criteria. Specifically we ask: (1) Is it possible to distinguish mast seeding species from high interannual variability in seed production? (2) To what extent is masting a prevalent strategy in a tropical community of Eastern South America?

## Materials and methods

### Study site

This study was conducted in a pristine tropical rainforest of Central French Guiana, at the Nouragues Biological Station (4°05 N, 52°40 W) [19]. Average annual rainfall is 2990 mm with a 2–3 mo. dry season, from September to November. The study area consists of two permanent study plots dominated by old-growth forest covering *ca.* 85 ha. One plot is on clay soil with a metamorphic volcanic substrate, and the other is on granitic-derived sandy soil. The flora of the Nouragues forest lists over 600 species of trees, shrubs and lianas [19], [20]. The species richness and biomass of ripe fruits, especially of animal-dispersed ones, peaks in April–May and is minimal in August–September [21].

### Data collection

Seedfall data were collected from a network of 160 seed traps set along a grid in an area of *ca*. 40 ha. Each trap consists in 0.5 m^2^ nylon-mesh nets, hung at 1.5 m above the ground to avoid disturbance from large mammals. Twenty traps were set up along five parallel trails on the clay soil plot (totaling 100 traps), and fifteen along four parallel trails on the sandy soil plot (totaling 60 traps) [22]. Minimum distance between nearest neighbour seed traps ranged from 15 to 50 m. Seeds and fruits were collected twice monthly since the establishment of the experiment, in February 2001. The present study reports results based on data collected until February 2006. All seeds, fruits, and fruit fragments >5 mm in size were identified to species or morphospecies. Seeds were categorized as mature (filled endosperm), partly eaten, or parasitized. In cases where fruits rather than seeds were counted, we used a mean seed per fruit ratio based on Van Roosemalen's published data [23], to convert the figure into a seed production. To avoid sampling from a single seed-bearing individual, we included only the species that had at least 50 recorded seeds in at least five seed traps. This way, we considered only the species for which we could be confident that our network of seed traps had received seeds from at least four different adults. We excluded small-seeded species as these usually passed through the mesh (*Cecropia, Ficus, Marcgravia, Miconia*), as well as species whose seeds could not be reliably identified to species or to a clear morphospecies (taxonomy follows Boggan *et al*. [24]). A total of 28 tree species, and 20 liana species were included in the study, the most abundant and fecund species at our site. Since these 48 species are distributed among 39 genera and 24 families, we did not expect a phylogenetic structure to bias our results.

### Statistical analyses

#### Autocorrelation and correlogram

For each species, *X_n_* is the total number of seeds of a given species found in seed traps at month *n*. The temporal autocorrelation of a time series is usually measured by 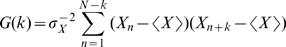, the ratio of the autocovariance to the variance *σ* ^2^ *_X_* of the time series *X*. Normalization was not an issue here since we were interested in capturing differences in seed production among species, and we did not seek to explore the variance around a mean trend, we used a non-normalized version of the above function, *C(k)*, defined as follows:
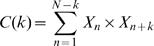
(1)Since seeds were collected over 60 months in our dataset, we let *k* vary between 0 and 29.

#### Modelling temporal patterns of seed production

Masting species should show a clustered pattern of seed production over time with the vast majority of the seeds falling in the same month or within a few consecutive months. Thus, masting species are expected to have high values of *C(0)* and a steady decay as the time lag k increases. *C(k)* could be modeled by a negative exponential function. In contrast, for species fruiting at yearly intervals, *C(k)* should be periodical. If the survey period increases, even masting species may eventually show quasi-periodic fruiting patterns, but the period would be much longer than annually fruiting species (typically>2 years).

For each species, we thus regressed *C*(*k*) against two models. Model 1 *(M1)* is a negative exponential distribution: 
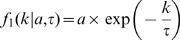
(2)where *a* is an estimator of *C*(*0*) and τ represents the mean temporal correlation time (in months): the smaller the τ, the shorter the fruiting season.

The second relationship *(M2)* models regular fruiting and is of the form: 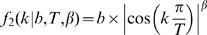
(3)where *b* is an estimator of *C*(0), similar to parameter *a* above, *T* represents the period of the fruiting pattern, and β is a shape parameter (high values of β yield peaked distributions of *C(k)*, while values of β close to zero yield flat distributions).

#### Parameter estimation

The best-fit parameter estimates were obtained by maximum likelihood inference. It is often assumed that for each species, the number of seeds fallen into seed-traps follows a Poisson distribution (uniform sampling). However, this does not directly inform us about the error structure of the autocorrelation function *C*(*k*). Here, we assume that the autocorrelation index *C(k)*, which is an integer number, also has a Poisson error distribution. Although we did not test this assumption directly, this is a reasonable choice in the absence of further information regarding the error structure of *C(k)*. For model 1, the likelihood of the model parameters, given the observed autocorrelation function *C*, was thus calculated as: 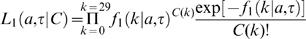
(4)and, for model 2, as: 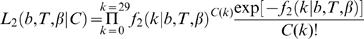
(5)


#### Model selection

The selection of the best model was based on a penalized likelihood measure of the goodness of fit, the Akaike Information Criterion (AIC), defined as twice the negative log-likelihood plus twice the number of parameters (4−2*L*_1_, for model 1, 6−2*L*_2_, for model 2) [25]. The best statistical model is the one with the lowest AIC. A common practical problem when implementing model-fitting procedures is that we may be trapped in local minima when exploring the parameter space to maximize the likelihood. To avoid this problem, we started from three independent initial values for each parameter and selected parameter combinations that minimized the AIC across all searches.

To illustrate the goodness of fit, we calculated the Pearson's *r^2^* correlation coefficient between model fits and observed data, following Dalling *et al.* [26]. We log-transformed the seed abundance records prior to all analyses in order to reduce deviations from normality.

#### Predictions

The AIC of the models, together with the inferred value of τ, the duration of a fruiting peak, and *T*, the fruiting periodicity, are all important for distinguishing between different reproductive strategies. Based on our 5-year dataset, we defined masting species as the ones showing one fruiting peak during the study period or two peaks separated by at least two years. Hence, we classified species as mast seeding in two cases: (1) model 1 is better than model 2 (AIC_M1_<AIC_M2_), and the length of the fruiting season is short (τ<6 mo); or (2) model 2 is better than model 1 and the fruiting periodicity is greater than two years (*T*>24 mo). This second condition is easily understood by realizing that in longer datasets, masting species will reveal irregular supra-annual fruiting peaks, and the *C(k)* function will be poorly modeled by an exponential function (model 1). Parameter *T* in model 2 will then represent the mean period separating supra-annual peaks, rather than a measure of periodicity. This condition does not imply that masting species should fruit in the same month each masting year. Here, we defined the temporal threshold distinguishing masting from non-masting species as 24 mo. Note however, that this threshold may be too conservative in longer-term studies. Also, documented masting species may occasionally fruit on two consecutive years (Y.-Y. Chen, pers. comm). Thus, our conditions defining a masting species may be too restrictive. It is only by examining very long-term datasets that we will be able to minimize this bias.

An annual fruiting species should be best fitted with model 2 and the fruiting periodicity *T* be close to 12 mo. Species that could not be classified into masting or annual categories were put into a third category, ‘irregular’ fruiting species.

To evaluate potential bias in previous attempts at identifying masting, we compared our results with predictions based on the CV index. The usual prediction is that the species showing high interannual variance in seed production (CV>1) are masting species.

All analyses were performed with the R statistical package, version 2.2.1 [27].

## Results

**Figure 1:**
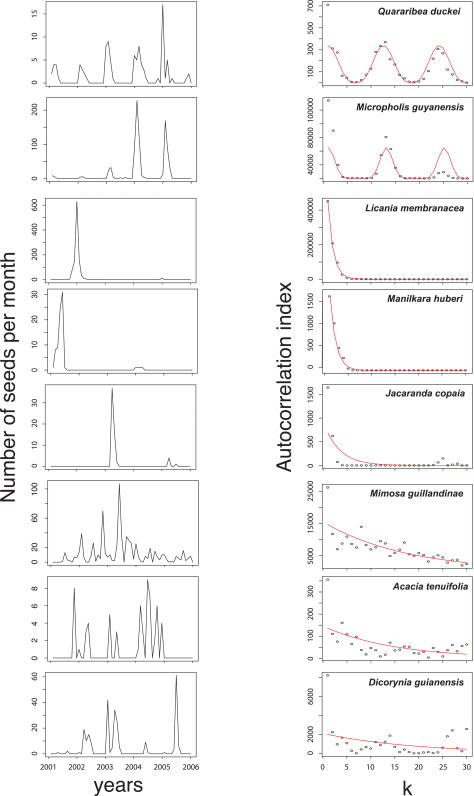
Left panels: observed seed production in the 2001–2006 period for 8 species, typical of the different fruiting strategies in the Nouragues forest. Right panels: observed and predicted values of the autocorrelation index *C(k)*.

Over five years, we collected and identified 11,699 seeds corresponding to the 48 study species (Table 1). This represented an average monthly seed fall of 2.44 seeds/m^2^/mo. Based on comparisons of models 1 and 2, and of the model parameters, we concluded that 26 species were consistent with an annual fruiting pattern (e.g., *Quararibea duckei*, Figure 1), 11 with a mast fruiting pattern (e.g., *Licania membranacea,* Figure 1), and 11 with an irregular pattern (e.g., *Mimosa guillandineae*, Figure 1) (Table 2). The 11 species classified as masting belong to nine different families, which are known to display other reproductive strategies. Thus, masting species were not phylogenetically clustered, and common ancestry cannot alone explain the high frequency of this strategy in our study. Estimates for the predicted decay time τ (exponential model) varied between 0.8 and 4.6 months in masting species. In the 11 irregularly fruiting species, estimates for τ varied between 6.4 and 22 months, and the temporal periodicity *T* was between 16.2 and 23.3 (periodic model) (Table 2). For 14 of our species, the inferred value of *T* was greater than 29 months, so the periodic model was not considered further. Estimates of the shape parameter *β* in the periodic model varied between 2.5 and 124.8, showing that the shape of the fitted curve varies widely.

**Table 1:** Taxonomic description of the study species, life form, dispersal syndrome, and number of sampled seeds.

Taxa	Family	Life form (dispersal syndrome)	No. seeds
*Acacia tenuifolia* (L) Willd.	Fabaceae	liana (AN)	62
*Aiouea guianensis* Aubl.	Lauraceae	tree (ZO)	306
*Arrabidaea* sp. 1	Bignoniaceae	liana (AN)	422
*Arrabidaea* sp. 2	Bignoniaceae	liana (AN)	68
*Bauhinia* sp. 1	Fabaceae	liana (BAL)	61
*Bombacoideae* sp. 1	Malvaceae	liana (AN)	60
*Combretum rotundifolium* Rich	Combretaceae	liana (AN)	87
*Cordia fulva* Johnston	Boraginaceae	tree (ZO)	118
*Couratari multiflora* (Smith) Eyma	Lecythidaceae	tree (AN)	83
*Dendrobangia boliviana* Rusby	Cardiopteridaceae	tree (ZO)	108
*Dicorynia guianensis* Amsh	Fabaceae	tree (AN)	250
*Doliocarpus paraensis* Sleumer	Dilleniaceae	liana (ZO)	63
*Fosteronia guyanensis* Müll Arg	Apocynaceae	liana (AN)	95
*Gouania blanchetiana* Miq	Rhamnaceae	liana (AN)	1169
*Guetarda acreana* Krause	Rubiaceae	tree (ZO)	920
*Hippocratea volubilis* L.	Hippocrateaceae	liana (AN)	219
*Inga alba* (Swartz) Willd.	Fabaceae	tree (ZO)	168
*Inga paraensis* Ducke	Fabaceae	tree (ZO)	196
*Inga thibaudiana* (Aublet) Don	Fabaceae	tree (ZO)	64
*Jacaranda copaia* (Aublet) Don	Bignoniaceae	tree (AN)	60
*Lacmellea cf. aculeata* (Ducke) Mon.	Apocynaceae	tree (ZO)	95
*Licania membranacea* Sagot ex J.M.A. de Lanessan	Chrysobalanaceae	tree (ZO)	1080
*Licania* sp. 1	Chrysobalanaceae	tree (ZO)	417
*Manikara huberi* (Ducke) Chevalier	Sapotaceae	tree (ZO)	78
*Mendoncia* sp. 1	Mendonciaceae	liana (ZO)	64
*Micropholis guyanensis* (A. D.C.) Pierre	Sapotaceae	tree (ZO)	846
*Mimosa guillandinae* (D.C.) Barneby	Fabaceae	liana (AN)	667
*Mimosa myriadenia* (Benth.) Benth.	Fabaceae	liana (AN)	930
*Moraceae* sp. 1	Moraceae	tree (ZO)	52
*Mouriri crassifolia*	Memecylaceae	tree (ZO)	102
*Ocotea floribunda* (Swartz) Mez	Lauraceae	tree (ZO)	131
*Paullinia cf. latifolia* Benth. ex. Radlk.	Sapindaceae	liana (ZO)	65
*Pourouma bicolor* Mart.	Urticaceae	tree (ZO)	94
*Pourouma tomentosa* Miq.	Urticaceae	tree (ZO)	396
*Protium cf. sagotianum* Marchand	Burseraceae	tree (ZO)	70
*Pseudopiptadenia suaveolens* (Miq.) Grimes	Fabaceae	tree (AN)	302
*Quararibea duckei* Huber	Malvaceae	treelet (ZO)	99
*Qualea rosea* Aubl.	Vochysiaceae	tree (AN)	84
*Qualea* sp. 1	Vochysiaceae	tree (AN)	309
*Rourea frutescens* Aublet	Connaraceae	liana (ZO)	61
*Serjania* sp. 1	Sapindaceae	liana (AN)	87
*Sterculia pruriens* (Aubl.) K. Schum	Malvaceae	tree (ZO)	56
*Stigmaphyllon* sp. 1	Malpighiaceae	liana (AN)	176
*Strychnos erichsonii* Rich. Schomb. ex Progel	Loganiaceae	liana (ZO)	265
*Tachigali melinonii* (Harms) Barneby	Fabaceae	tree (AN)	164
*Tetrapterys* sp. 1	Malpighiaceae	liana (AN)	88
*Tetrapterys* sp. 2	Malpighiaceae	liana (AN)	286
*Virola michelii* Heckel	Myristicaceae	tree (ZO)	56

**Table 2:** Fitted parameters (a, τ, *b*, *T*, β), AIC values of the two models predicting temporal autocorrelation in seed production along 30 months for each species, Pearson regression coefficient of observed against predicted values, and CV.

Taxon	Model 1: Negative exponential				Model 2: Sinusoid					Strategy	CV
	*A*	τ	AIC	r^2^	*b*	*T*	β	AIC	r^2^		
*Acacia tenuifolia*	136.6	14.7	**969**	0.21	-	-	-	-	-	Irregular	1.18
*Aiouea guianensis*	2961.3	21.4	62992	0.06	3245.1	13.0	2.5	**38769**	0.34	Annual	0.83
*Arrabidaea* sp. 1	26768.2	4.6	**313524**	0.18	15058.3	10.9	6.4	326757	0.35	Masting	1.8
*Arrabidaea* sp. 2	444.2	6.5	8027	0.05	343.4	16.2	5.2	**7198**	0.20	Irregular	1.13
*Bauhinia* sp. 1	440.3	4.9	4755	0.31	344.2	16.3	9.1	**3966**	0.31	Irregular	1.7
*Bombacoideae* sp. 1	193.4	10.5	2394	0.17	166.8	11.6	3.5	**1523**	0.73	Annual	0.86
*Combretum rotundifolium*	204.2	23.7	4291	0.01	312.8	12.1	4.4	**863**	0.90	Annual	0.59
*Cordia fulva*	503.9	17.9	14794	0.03	571.3	11.5	2.7	**10064**	0.10	Annual	1.32
*Couratari multiflora*	3871.5	1.1	**8067**	0.42	-	-	-	-	-	Masting	1.87
*Dendrobangia boliviana*	332.0	22.0	**5392**	0.03	-	-	-	-	-	Irregular	0.91
*Dicorynia guianensis*	1990.4	19.2	**37635**	0.05	-	-	-	-	-	Irregular	1.08
*Doliocarpus paraensis*	135.9	18.9	3486	0.02	156.9	26.0	4.0	**1959**	0.35	Masting	1.15
*Fosteronia guyanensis*	702.4	7.8	14494	0.02	1073.5	12.2	25.2	**4989**	0.88	Annual	1.54
*Gouania blanchetiana*	79028.2	10.3	1027132	0.01	49571.3	12.5	3.9	**467749**	0.66	Annual	0.55
*Guetarda acreana*	34475.0	14.9	431429	0.04	31845.5	11.9	3.0	**132223**	0.81	Annual	0.6
*Hippocratea volubilis*	2642.0	10.2	30411	0.21	2172.3	12.3	3.8	**20975**	0.46	Annual	1.5
*Inga alba*	3118.1	6.2	66510	0.04	4107.0	11.7	25.8	**36801**	0.50	Annual	1.83
*Inga paraensis*	2613.9	10.0	88705	0.04	7502.6	13.1	80.6	**17996**	0.57	Annual	0.78
*Inga thibaudiana*	108.2	25.3	2960	0.00	205.3	12.2	6.9	**405**	0.97	Annual	0.64
*Jacaranda copaia*	684.2	3.3	5780	0.05	745.7	24.3	92.0	**3067**	0.76	Masting	1.18
*Lacmellea aculeata*	889.6	5.6	10928	0.10	668.2	12.4	9.9	**9284**	0.54	Annual	1.51
*Licania membranacea*	452012.5	1.1	**14588**	0.95	-	-	-	-	-	Masting	1.67
*Licania* sp. 1	30209.2	2.5	**21509**	0.55	-	-	-	-	-	Masting	1.38
*Manikara huberi*	1928.7	1.3	**346**	0.86	-	-	-	-	-	Masting	2.1
*Mendoncia* sp. 1	103.2	19.7	1724	0.01	124.3	12.3	3.3	**657**	0.88	Annual	0.68
*Micropholis guyanensis*	54019.3	7.5	644305	0.21	45048.3	12.1	8.7	**350008**	0.49	Annual	1.35
*Mimosa guillandinae*	14668.1	17.6	**20745**	0.76	-	-	-	-	-	Irregular	0.95
*Mimosa myriadenia*	67419.0	6.4	**204367**	0.76	-	-	-	-	-	Irregular	1.5
*Moraceae* sp. 1	1143.9	1.5	4121	0.21	880.4	1.7	122.0	**3318**	0.35	Annual	1.09
*Mouriri crassifolia*	419.1	13.6	9384	0.02	597.1	11.9	7.7	**3038**	0.92	Annual	1.03
*Ocotea floribunda*	2405.1	3.5	**15998**	0.61	-	-	-	-	-	Masting	0.9
*Paullinia cf. latifolia*	133.0	19.2	3336	0.01	147.2	12.4	2.8	**2234**	0.30	Annual	0.7
*Pourouma bicolor*	294.4	17.9	3184	0.10	271.1	12.5	1.9	**1896**	0.57	Annual	0.79
*Pourouma tomentosa*	6770.9	12.6	162815	0.00	11863.1	11.8	14.0	**26898**	0.89	Annual	0.79
*Protium cf. sagotianum*	283.6	10.1	6301	0.00	327.7	11.9	7.8	**3483**	0.71	Annual	1.41
*Pseudopiptadenia suaveolens*	11569.2	4.4	99659	0.21	7631.8	12.0	12.4	**89241**	0.65	Annual	1.47
*Quararibea duckei*	254.3	22.4	4244	0.09	340.4	11.6	3.1	**918**	0.89	Annual	0.44
*Qualea rosea*	3959.5	0.8	**232**	0.91	-	-	-	-	-	Masting	0.17
*Qualea* sp. 1	6875.1	9.0	215554	0.01	21533.9	23.3	124.8	**21058**	0.92	Irregular	0.11
*Rourea frutescens*	121.1	16.2	1834	0.06	140.0	11.3	3.2	**607**	0.83	Annual	0.98
*Serjania* sp. 1	470.1	9.1	**4298**	0.25	302.1	12.3	2.5	4420	0.36	Irregular	1.34
*Sterculia pruriens*	1187.3	1.6	**3346**	0.42	-	-	-	-	-	Masting	2.13
*Stigmaphyllon* sp. 1	768.0	25.3	13737	0.02	1059.7	11.9	3.1	**3422**	0.80	Annual	0.69
*Strychnos erichsonii*	3063.7	13.4	**28116**	0.17	1931.5	13.0	1.1	31684	0.17	Irregular	1.34
*Tachigali melinonii*	1870.1	7.9	**9182**	0.76	-	-	-	-	-	Irregular	1.11
*Tetrapterys* sp. 1	301.5	17.2	7173	0.01	370.5	8.7	4.1	**3767**	0.53	Annual	1.31
*Tetrapterys* sp. 2	31915.5	1.2	**93330**	0.19	-	-	-	-	-	Masting	1.81
*Virola michelii*	109.2	15.9	3145	0.03	143.0	6.1	4.4	**1769**	0.60	Annual	0.93

Boldface indicates the best model for each species. If the periodicity *T* in model 2 was greater than *k*_max_ = 29 months, then model 2 was assumed non-biological and was not further considered as an option.

The predictive power of our models varied significantly across species (range: 0.03–0.97), but it was generally high (Table 2). In 29 of the 48 species, the Pearson *r* ^2^ was greater than 0.5, and among these, 20 had a *r* ^2^ greater than 0.7. Variation in the goodness of fit is a reflection of the complexity of the temporal patterns in seed production. The poorest fits were found for taxa with an irregular fruiting pattern (e.g., *Dendrobangia boliviana* and *Dicorynia guianensis*), and the best fits for taxa showing clear annual or masting patterns (e.g. *Pourouma tomentosa* and *Licania membranacea*, respectively).

We found that 28 of the study species (58%) had a CV>1. The match between our classification and that based on the CV was just over a half (52%, 25 species). Among the species with CV>1, ten (36%) were classified as annual fruiters with the maximum likelihood method (*Cordia fulva, Fosteronia guyanensis*, *Hippocratea volubilis*, *Inga alba*, *Lacmellea cf. aculeata*, *Micropholis guyanensis*, *Mouriri crassifolia*, *Protium sagotianum*, *Pseudopiptadenia suaveolens*, *Tetrapterys* sp. 1). These species showed important interannual variability in seed production leading to a high CV index, but they all had an estimated periodicity of *ca.* 12 mo.

One taxon, *Moraceae* sp. 1, was classified as annually fruiting but this characterization of its reproductive strategy was not satisfactory. The absence of seed production during 2001, 2002, 2003 originally suggested that this is a supra-annual fruiting species, yet the estimated periodicity was of *T* = 1.7. This does not correspond to the observed seed production pattern, and is not biologically meaningful as this would mean that this taxon fruits intermittently every two months.

We found evidence of a differential reproductive strategy between trees and lianas. Of the 11 masting species eight were trees, and only three were lianas (*Arrabidaea*, *Doliocarpus*, *Tetrapterys*). Of the eight masting tree species, six were tall shade-tolerant species (in genera *Couratari*, *Licania*, *Manilkara*), and two were tall light-demanding species (*Qualea*, *Sterculia)*. Most animal-dispersed trees fruited annually (16/25). However, most lianas were anemochorous and showed an annual or irregular fruiting pattern (17/20).

## Discussion

### Characterization of the reproductive strategies

Using an objective set of quantitative criteria, we were able to differentiate mast seeding species from high interannual variability in seed production for 48 woody species of an old-growth tropical rain forest in French Guiana. Almost a quarter of the studied species showed masting patterns. Previously published flowering and fruiting phenological classifications were based on the description of the phenology of a limited number of species, and they did not encompass the vast array of reproductive strategies displayed by tropical plants [28]–[31]. In an attempt to obtain a better description of phenological variation in tropical forests, Newstrom *et al*. [32] proposed a commonly used classification defining four classes of fruiting strategies: continuous, sub-annual, annual and supra-annual. However, their classification clearly lacked a quantitative basis. In part to circumvent such limitation, several authors have used the coefficient of variation in interannual seed production to distinguish among reproductive strategies. While this has been a major improvement, the CV still overlooks obvious differences in reproductive strategies because it measures year-to-year variation in seed output, a characteristic that does not always distinguish among different seed production patterns. The current study is one of the few to provide robust estimates of the frequency of masting species in a tropical forest.

All three reproductive strategies defined in this study show, to some extent, year-to-year variation in seed output. Annual species displayed a regular and periodic pattern, but many species showed large fluctuations in seed production across years (e.g. *Micropholis guyanensis*, Figure 1). Among irregularly fruiting species, 3 out of 11 fruited continuously throughout the study period with a marked peak of fructification (e.g., *Mimosa guillandinae*, Figure 1). The other eight species showed an erratic pattern with fruiting episodes occurring at any time of the year, and varying greatly in duration (e.g. *Acacia tenuifolia*, Figure 1). Thus, what we here referred to as the irregular reproductive strategy groups species with high interannual variability, but that are clearly distinct from either the masting and the annual strategies. Overall, high interannual variability in seed production was a frequent pattern among woody species in this tropical forest.

### Regional comparisons

ter Steege and Persaud [33] reported monthly fruiting and flowering data for 960 trees belonging to 190 species over 12 years (1942–1954) at the Mazurini Station in Guyana, a locality that is geographically close to Nouragues. The data from Mazurini is consistent with our findings for most species shared between the two studies. *Jacaranda copaia*, *Couratari multiflora* and *Sterculia pruriens* showed supra-annual fruiting at Mazurini: all three species fruited only twice between 1942 and 1954, and they were classified as masting species in our study. *Manilkara huberi* showed a strong masting pattern at Nouragues and *Manilkara bidentata* fruited three times over the 12-year study period also suggesting that this is a mast fruiting species. These two species occur in sympatry at Nouragues and previous research suggests that both show masting patterns [34]. *Eperua falcata* and *Inga thibaudiana* showed annual fruiting patterns at both Nouragues and Mazurini. Only one species, *Inga alba,* showed contrasting phenological patterns in the two sites: this species showed high interannual variability in seed production at Nouragues but fruiting was reported only once at Mazurini.

Differences in climatic variables may be the source of discrepancies in phenological patterns between sites if a species phenology is strongly dependent on local environmental cues. At larger geographic scales, such differences may be more conspicuous [35], [36]. A remarkable example is provided by *Jacaranda copaia,* a tree species with a wide range distribution across Central and South America. Unlike at Mazurini and at Nouragues, *J. copaia* is not reported as a masting species at Barro Colorado Island in Panama [37], [38]. This suggests that a species may show quite different reproductive strategies among regions, arising from species-specific responses to local cues driving phenological patterns. Such patterns may have critical consequences in recruitment success. Surprisingly, we are unaware of another example of a species recorded as masting in one area and as a regular fruiter in another place, like *J. copaia*. A long-term network of monitoring sites for fruiting patterns in the neotropics would be useful to describe spatial variation in seed production and to examine how it may be influenced by climate variables.

### Masting in tropical communities

Kelly and Sork [1] hypothesized that tropical forests have lower interannual variation in seed production than temperate forests. While we do not directly test this hypothesis, we suggest that the different forces driving mast seeding in temperate forests are likely to operate in tropical forests as well. First, we showed that the CV index does not make a clear distinction between species showing mast seeding and high interannual variation in seed production. This can lead to an overestimation of the occurrence of masting. Since the conclusions of all temperate studies are based on the CV index, the prevalence of mast seeding at high latitudes might be lower than previously thought. Second, the frequency of masting, as obtained in several studies based on literature surveys [1], [13], may reflect phylogenetic conservatism in groups such as Fagaceae and Pinaceae. In our study we found masting species in no less than nine different families that include species displaying other reproductive strategies. Thus, the number of independent evolutionary changes towards masting may be much higher in tropical than in temperate forests.

Theories aiming at explaining the evolution of masting have emphasized its role as a mechanism for predator satiation and increased pollination efficiency in wind-pollinated species [1]. These processes are likely to play a role in both tropical and temperate forests. While several studies showed evidence that predator satiation is an important factor in driving mast seeding in many tropical tree species [11], [39]–[41], we were not able to find any information on the possible role of masting in increased pollination efficiency in the tropics. Interestingly, recent work has revealed that mast seeding could also be associated to the presence of ectomycorrhiza [42]–[44]. There are many examples of masting in temperate ectomycorrhizal families such as Fagaceae and Pinaceae, but these associations also prevail in masting tropical families such as the Dipterocarpaceae [45] and the Fabaceae [42], [44], [46]. Unfortunately ectomycorrhizal associations are poorly known from South-American tropical forests, and data on those of our most clearly masting species (*Licania* and *Manilkara*) are currently lacking.

Our study shows a high proportion of mast seeding species. At the Nouragues forest, masting species represented almost a fourth of the study species, while just over a half showed a clear annual fruiting pattern (54%). This finding is consistent with those obtained in a tropical forest of central Panama [5] and in the tropical dipterocarp forests of South-East Asia [10], [47]. However, other tropical community-level studies have reported much lower frequencies of mast seeding. For example, a 4-year study of the fruiting phenology in a tropical mountain forest of the Philippines found only three species out of 34 showing supra-annual fruiting, that is, less than 10% [48]. Newstrom *et al*. [32], in a 12-year survey of flowering for 173 species at La Selva Biological Station in Costa Rica, found that less than 9% of the studied species had a supra-annual flowering pattern.

If the prevalence of mast seeding in French Guiana is unusually high for the tropics, this could be due to a regional climatic effect. Indeed, there is evidence that strong picks of seed production are related to El Niño Southern Oscillation events both in South-East Asian dipterocarp forests [11] and in Panamanian forests [49]. It was found that the Guianas exhibit a strong El Niño anomaly, causing widespread droughts during El Niño years [50], [51]. However, the masting events observed during the study period did not coincide with El Niño years, making the relation between mast years and El Niño equivocal. Our 5-year study may just be too short to observe emergent patterns linking mast years and El Niño events, and does not allow to clearly conclude about the influence of climatic cycles in fruiting phenology in this region. Long-term flowering and fruiting data combined with modelling approaches will be necessary for a better understanding of the factors determining mast fruiting.

### Conclusions

We provided evidence that a significant fraction of the tree and liana community in a forest of French Guiana shows masting patterns, and suggest that the prevalence of such massive events in tropical communities could have been overlooked. Generalisations about the factors determining mast seeding will only become possible through an increased effort in long-term monitoring of phenological patterns in the tropics. Plant species appear to benefit from their large seed crops at irregular intervals, relative to annually fruiting species [5]. Thus, the wide occurrence of masting species in tropical plant communities may play a role in diversity maintenance through storage dynamics. Since the onset of masting is partly related to climate variables [10], [52], such dynamics is important to document with greatest accuracy in the context of global climate change. This could help to improve the accuracy of predictions of plant population shifts under global change scenarios.
